# Montessori Principles in Medical Education: Training Innovative and Compassionate Physicians

**DOI:** 10.1007/s40670-026-02705-8

**Published:** 2026-03-26

**Authors:** Pamela Meyers, Sandra Guzmán-Figueroa, Ryan Patterson, Dina Kurzweil, Mabel Perez-Oquendo, Maheshinie Rajapaksha, Devi Rajan, Renee J. Chosed

**Affiliations:** 1 Teaching and Learning Center, Oregon Health & Science University, Portland, OR USA; 2 Curriculum Development & Innovation Office, Academic Deanship, Universidad Central del Caribe, Bayamón, Puerto Rico; 3 Education and Technology Innovation Support Office, Uniformed Services University of the Health Sciences, Bethesda, MD USA; 4 Student Opportunities for Advancement in Research Office, Baylor College of Medicine, TX Houston, USA; 5 Department of Biomedical Sciences, Mercer University School of Medicine, Savannah, GA USA; 6 Department of Biomedical Sciences, University of South Carolina School of Medicine Greenville, 701 Grove Rd, SC 29605 Greenville, USA

**Keywords:** Montessori method, Self-directed learning, Experiential learning, Lifelong learning

## Abstract

The Montessori Method emphasizes competency-based assessment, student-directed learning, and teachers as guides who help learners become reflective, lifelong professionals. This paper examines how Montessori principles inform medical education, promoting connections between foundational learning and patient-centered care. The authors developed the Montessori-Influenced Medical Education (MIME) Framework to classify and align these principles with medical school programs, revealing their implicit presence throughout training. Elements such as self-directed learning, experiential practice, and interprofessional collaboration mirror Montessori values. Thoughtful integration of individualized pathways and mastery learning can foster adaptable, resilient, compassionate healthcare professionals equipped to meet the evolving challenges of modern patient care.

## Introduction

The Montessori Method of education, established by Dr. Maria Montessori in the early 20th century, is recognized for its child-centered approach, emphasizing self-directed learning, sensory education, and the prepared environment as a foundation for lifelong learning. Traditionally applied to early childhood and elementary education, the Montessori Method effectively fosters critical thinking, independence, and intrinsic motivation in children [1, 2]. As contemporary medical education seeks to cultivate adaptable, empathetic, lifelong learners equipped to address evolving healthcare challenges [3], there is growing interest in leveraging the principles of the Montessori Method to inform curriculum design to foster a more active, self-directed learning experience for medical students.

Originally designed to support young children’s developmental needs, Maria Montessori’s principles offer significant potential for application for adult learners in medical education. The Montessori Method of education, with its focus on autonomy and self-regulated learning, may initially seem incompatible with the structured, lecture-heavy curriculum of the pre-clerkship years of medical school. The transition into clinical clerkships marks a natural pivot, for it is in the immersive, patient-centered world of the hospital wards and clinics that the medical students learn through observation and feedback that is embedded within the Montessori Method.

From the third year forward, medical education moves away from a primarily lecture-based approach and transforms into a model of experiential, self-regulated learning. Students are no longer passive recipients of information [4] and are responsible for their own learning, driven by the needs of their patients. This aligns directly with Montessori’s emphasis on fostering independence and intrinsic motivation. Faculty roles also shift dramatically, moving from lecturer to guide. Attending physicians and residents mentor and coach students, facilitating inquiry and refining clinical reasoning. This practical application of knowledge helps students develop critical thinking and self-assessment skills, building authentic professional relationships in the process [5]. The fundamental clinical practice of observation aligns with the Montessori perspective, which highlights the value of setting aside personal biases and assumptions in order to better understand reality as it is [6].

This paper first performs a literature review to identify key Montessori principles. Because “Montessori” is an unregulated term [7, 8], this paper focuses on broadly accepted principles of the Montessori Method and examines their integration into medical education. Table 1 defines the key principles of the Montessori Method. We then explore how Montessori principles may be reflected, both implicitly and effectively, across various phases of medical education. Finally, we map these principles onto the existing educational practices found in all phases of medical education and present a framework that supports greater intentionality and coherence to the entire medical school curriculum. Identifying and integrating Montessori principles into medical education is not about replacing existing components but rather optimizing them to create a more cohesive and learner-centered approach that empowers students to become adaptable, compassionate, life-long learners who can meet the demands of a dynamic, patient-centered healthcare environment. Because medical students are adult learners, we also situate Montessori principles within established adult learning theories to clarify their relevance to this context.

**Table 1:** Definitions and examples of various terminologies used in Montessori Method-Based Medical Education in our Montessori-Influenced Medical Education Framework (MIME Framework):

Model Components		Definitions	Examples
Structure		The features and infrastructure of a medical school that support the educational mission (LCME, 2025).	● Simulation centers ● Libraries and digital learning resources ● Virtual reality (VR) ● Entrustable Professional Activities (EPAs) ● Student-run clinics
Process	Curriculum	The components of the delivery of the educational program.	● Problem-Based Learning (PBL) ● Team-Based Learning (TBL) ● Flipped classrooms ● Interprofessional education ● Integration of technology (e.g., telemedicine)
	Freedom	The term freedom is used in a variety of ways in medical literature, including academic freedom, patient autonomy, and freedom of choice in career. For our purposes, we will focus on freedom as autonomy regarding how students learn, specifically, that students “behave with feelings of volition, willingness, and choice.”	● Elective selection ● Flexible learning pathways ● Independent study options ● Choice of instructional modality (e.g., in-person vs. recorded sessions)
Outcomes		The learner assessments used to measure competency mastery. This category was missing from the Fidelity Model, but present in the Logic Model, and was needed as assessments are an essential component of medical education	● Objective Structured Clinical Examinations (OSCEs) ● Board exams

Scholarship in medical education often draws on frameworks such as self-determination theory and competency-based medical education (CBME). By comparison, Montessori education offers a complementary framework with its own distinct theoretical foundations. Self-determination theory focuses on motivation, and CBME organizes outcomes and assessment. Montessori, in contrast, emphasizes intentionally designing learning environments that support autonomy, meaningful work, increasing responsibility, and supported independence. Montessori principles emphasize thoughtfully designed learning environments, careful attention to learner readiness, and teachers who act as guides which brings together motivation, structure, and growing independence in a practical way. Therefore, Montessori principles do not replace existing frameworks but offer a unifying perspective on how learning contexts can be structured to support self-directed, experiential development throughout a student’s medical training.

The following section outlines the foundational principles of the Montessori Method, providing context for their application in medical education.

### Defining the Montessori Method in the Context of Medical Education

Montessori education is a distinctive approach inspired by Dr. Maria Montessori’s educational practices. Three key elements that serve as the foundation of the Montessori Method are the prepared environment, the teacher, and the student [8, 9]. The prepared environment supports learner development through learner choice, self-directed learning, and learning materials [7, 9]. These materials allow students to explore sensory and academic concepts by engaging hands-on with the content, setting their own pace and choosing their own activities [9]. Self-directed learning involves students controlling the learning process, including determining learning objectives, identifying resources, implementing strategies, and evaluating their learning outcomes [10, 11]. The teacher, meanwhile, shifts positions from the giver of knowledge to a guide [12], moving toward a learner-centered model that is responsive to students’ unique needs, interests, and learning paces [13]. Teachers and students then work to create an environment that supports students’ needs and development [12, 14]. Other key elements of the Montessori Method include an interactive learning environment, active learning, and uninterrupted work cycles [12].

The Montessori Method has gained international recognition in recent years but is still often characterized as an alternative pedagogical approach, existing “on the margins” of mainstream educational practices, with minimal focus on how the principles could be applied to adult learners [7, 8, 15]. Given this limited recognition, this study investigated how the Montessori Method may inform medical education by exploring how its core principles could be incorporated into existing medical school curricula to strengthen medical student training and improve educational outcomes. The methodological framework employed in this study is detailed in the subsequent section.

## Methodology

### Literature Search

To explore the presence and integration of Montessori principles within medical education, a multidisciplinary working group conducted a structured narrative literature review to determine whether elements of the Montessori Method have been incorporated into contemporary medical school curricula. As a structured narrative review rather than a formal systematic review, this study may not have captured all relevant literature. While efforts were made to use iterative search strategies and multidisciplinary screening, the framework derivation reflects interpretative synthesis rather than quantitative meta-analysis.

Searches were conducted in PubMed, ERIC, and Google Scholar to capture the evolution of competency based and learner-centered reforms in medical education. We limited inclusion of publications that were from 1997 to 2025, English-language sources, peer-reviewed empirical, conceptual papers, and relevant literature such as policy reports, institutional white papers, among others. Approximately 160 sources were screened, and 136 were included.

Titles and abstracts were independently screened by members of the working group and full-text review was conducted for sources meeting inclusion criteria or when relevance was unclear. Discrepancies were resolved through consensus discussion.

Through collaborative discussion, the working group identified core Montessori principles and translated them into search keywords derived from both Montessori education literature and medical education scholarship.

Using keywords from the principles of the Montessori Method, we used the following search terms: *Learner-centered*,* hands-on learning*,* prepared environment*,* self-directed learning*,* respect for learner*,* professionalism*,* uninterrupted work cycle*,* role of the teacher*,* educating the whole learner*,* nature and reality*,* practical life skills*,* prepared learning environment*,* integration of subjects*,* mixed levels of learners/education*,* competency-based*,* integration of technology*,* critical thinking*,* student choice*,* respect for unique backgrounds*,* research and innovation*,* and life-long learner*. Each core search term was combined with medical education qualifiers (e.g. medical school curriculum, health sciences curriculum, pharmacy education, undergraduate medical education and simulation) to refine the results. These qualifiers were used strategically depending on database syntax and the focus of individual search queries. This approach helped identify literature where aspects of the Montessori Method appeared (or did not appear) in medical education contexts. In cases where exact term matches did not yield results, we used alternative terminology more relevant to medical education to capture literature discussing analogous concepts related to the Montessori Method. For example, we combined *holistic* with *medical school curriculum* when searching for the principle of *educating the whole learner.* Other alternative terminology included *project-based learning*,* team-based learning*, and *flipped classroom* for *learner-centered; simulation* for *hands-on learning*; *learning space*, and *technology integration* for *prepared environment; IPE undergraduate medical curriculum* for *mixed levels of education; simulation undergraduate medical education* and *EMT training medical school* for *nature and reality* and *practical life*; *life skills*,* employability skills*,* competence*, and *transferable skill* for *practical life;* and *humanities* and *undergraduate medical education curriculum* for *integration of subjects.* This iterative strategy was designed to identify literature in which Montessori-aligned principles appeared, even when not labeled explicitly as Montessori.

### Development of the MIME Framework: Montessori-Influenced Medical Education Framework

Due to the lack of a single verified source outlining all Montessori principles, we identified a model used by Montessori education researchers. We first reviewed the Logic Model for Montessori Research [16], which organizes the Montessori core components in the headings of “Inputs/Resources,” “Activities/Actions,” “Outputs,” “Outcomes,” and “Impacts.” We attempted to organize the examples of Montessori principles we identified in medical education under each heading. However, we encountered challenges in distinguishing among “Outputs,” “Outcomes,” and “Impacts,” as these often overlapped in the context of medical education. We then reviewed a Montessori fidelity model which organizes Montessori practices into two main components: *structure* and *process. Process* is further broken down into *curriculum* and *freedom* [17]. After reviewing the fidelity model, we decided to add “Outcomes” as an additional component for our analysis, as originally presented in the Logic Model [16]. Informed by the Liaison Committee on Medical Education [18] standards and the fidelity model [17], we refined our components to create the Montessori-Influenced Medical Education Framework (MIME Framework) (Table 1). Although adult learning theory is not represented as a separate component in the MIME framework, its principles provided contextual grounding for applying Montessori principles within the context of medical education.

## A New Paradigm

**Fig. 1:**
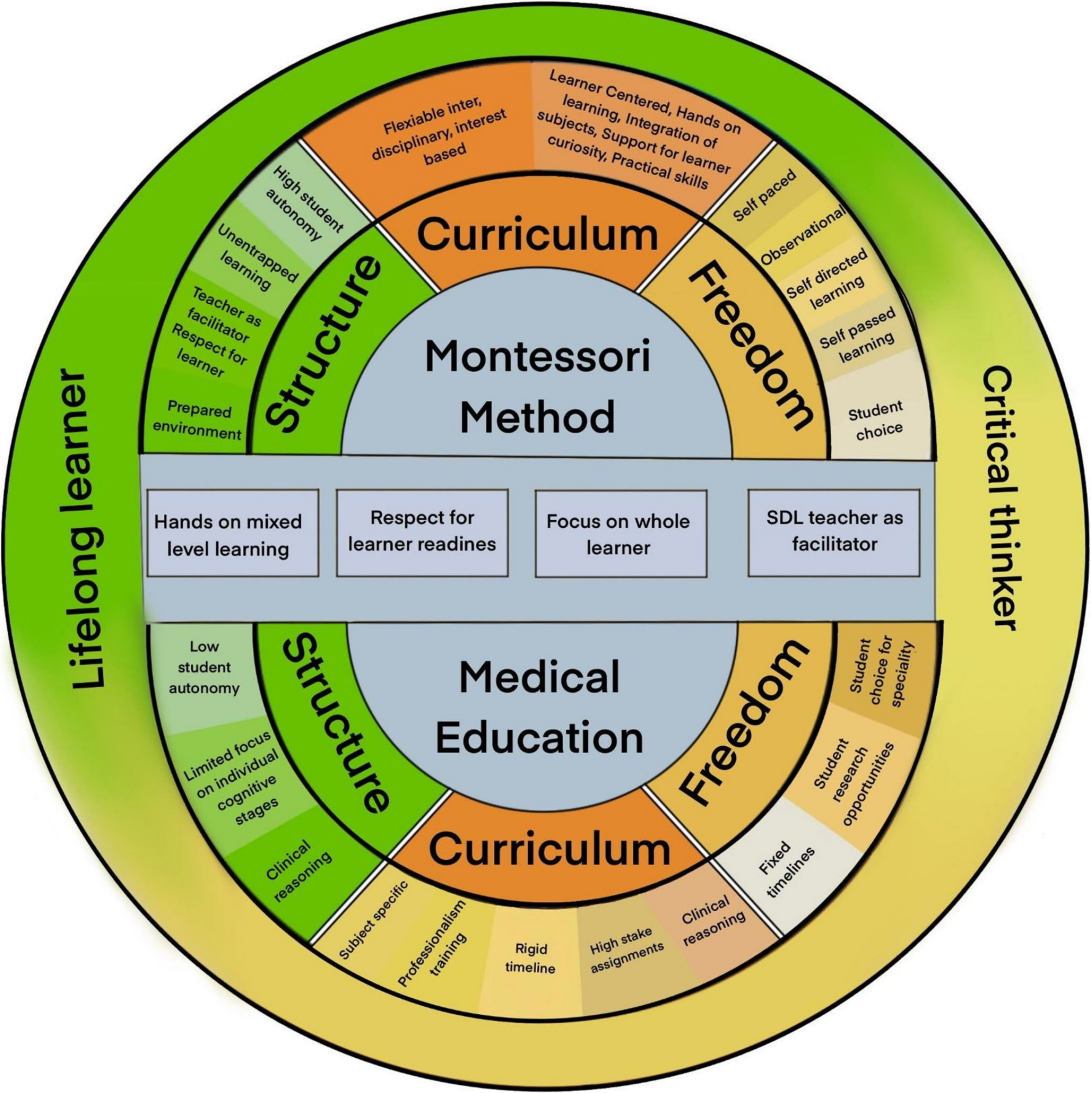
Organization and assignment of medical school curricular items to the Montessori Method within the MIME Framework

Based on education models from the Montessori Education Literature, we created the MIME Framework. This framework categorizes and assigns medical school curricular items to the Montessori Method as shown in Fig. 1. Table 2 provides a structured approach for applying the MIME Framework in practice.

**Table 2:** Applying the MIME Framework

Steps	Action	Example	Measures/Indicators
Step 1 Select Unit	Identify a course or curricular phase	Biochemistry block Choose a course, block, session, or clinical learning activity (e.g., “pre-clerkship biochemistry block,” “cardiac auscultation lab,” “ambulatory clinic half-day”).	Course objectives; syllabus review
Step 2 Map to MIME	Map learning objectives to **Structure**,** Process (Curriculum and Freedom)**,** and Outcome**s	Lectures (Structure); flipped classroom (Process – Curriculum); elective selection (Process – Freedom); exam performance (Outcomes) Structure: environment, materials, time blocks, grouping, faculty roles, assessment infrastructure Process: learning activities and routines (cases, labs, simulations, conferences, reflection) Curriculum/Freedom: where autonomy is supported (choice, pacing, self-assessment, role-taking, peer teaching) and where it is constrained Outcomes: target competencies (knowledge/skills/professional identity) and evidence sources	Curriculum mapping tool; session observation; metrics and indicators
Step 3 Identify gaps and misalignment	Identify gaps Identify where intended outcomes are undermined by design features	Low autonomy; limited uninterrupted work time Low autonomy support, limited uninterrupted work time, “one-size-fits-all” sequencing, weak feedback loops, few peer-teaching opportunities).	Autonomy-support measures; reflective survey responses
Step 4 Measure + Redesign using MIME aligned strategies and metrics	Redesign strategies and define suggested metrics Implement targeted changes and select feasible indicators to monitor impact.	Incorporate protected time for self-directed learning; introduce coaching checkpoints (e.g., longer protected practice blocks, choice of cases, structured peer coaching, formative feedback routines)	Reflective writing analysis; competency progression indicators, redesign

*Iterative evaluation & Continuous Improvement*

Component 1: Structure.

The Structure component in the framework refers to the infrastructure that supports a medical school’s educational mission (e.g., learning environment, time allocation, faculty roles and coaching/mentoring systems). The educational framework for allopathic medical schools in the United States is established through standards defined by the Liaison Committee on Medical Education [18].

The Montessori Method emphasizes a structured yet flexible learning environment, paralleling the problem-based learning (PBL) classrooms common in medical education. Both Montessori and medical education models further emphasize access to well-equipped learning centers (including libraries and digital resources) and the integration of modern technologies such as virtual reality (VR) [19–22]. A central tenet in both systems is respect for the learner, reflected in the professionalism and ethical standards reinforced through Entrustable Professional Activities (EPAs) as outlined by the Association of American Medical Colleges (AAMC) [19, 23–26]. In both models, teachers function as facilitators who promote learner autonomy while cultivating a collaborative and supportive educational environment [27–33]. Both educational paradigms emphasize reality-based, experiential learning as a means of fostering critical thinking and practical competence. In medical education, this manifests through simulation centers, community health initiatives, and emergency medical training, providing opportunities for authentic engagement and skill application [34–39]. Similarly, both systems prioritize educating the whole learner by nurturing empathy, emotional well-being, and a sense of belonging, often through community service and interdisciplinary collaboration [39–45].

Component 2: Process.

The process components of our framework encompass the delivery (instructional strategies) and experience (learner activities) of the educational program, represented by Curriculum and Freedom.

A. Curriculum.

The medical education curriculum embodies a learner-centered philosophy through diverse pedagogical methods, including PBL, TBL, and flipped classrooms. Experiential learning is supported through student-run clinics and simulation-based training, while self-directed learning fosters independent inquiry. Interprofessional education enables collaboration across health disciplines, and technological integration [including virtual and augmented reality, digital health platforms, and telemedicine) further enriches the learning process [14, 46–52].

B. Freedom.

Freedom, another foundational Montessori principle, appears in medical education through self-directed learning and curricular flexibility. Students personalize their academic trajectories by selecting electives, choosing instructional modalities, and engaging in research and innovation in the clinical environment. Inclusivity and cultural humility are also prioritized to support learners from diverse backgrounds [10, 11, 53–57].

Component 3: Outcomes.

The Outcomes component of our framework addresses learner assessments that measure competency mastery, specifically those related to competency attainment, reflective capacity, professional identity formation, and performance indicators. Both Montessori and medical education models emphasize mastery learning and individualized progression grounded in continuous reflection. In Montessori settings, assessment occurs through sustained observation, self-correction, and iterative feedback rather than isolated examinations. This approach parallels competency-based medical education, where learners advance through formative assessment and direct observation of performance over time.

Within the MIME Framework, outcomes serve as a feedback loop connecting **structure** and **process.** Ultimately, both Montessori and medical education frameworks share a commitment to lifelong learning, competency-based education, and individualized progression. Table 3 illustrates some examples of where Montessori Principles are applied in early education and medical education. The mastery of clinical and professional skills is objectively assessed through methods such as Objective Structured Clinical Examinations (OSCEs) and board certifications, reinforcing the continuous development of competent, reflective, and ethical practitioners [58–64].

**Table 3:** Examples of how Montessori Principles can be applied to early childhood education and medical education

Montessori Principle	Early Childhood Education Example	Medical Education Example (MIME Framework)
Prepared Environment	A classroom equipped with child-sized furniture and accessible shelving where a child can independently select stencils and paper to practice writing letters.	A simulation lab or student-run clinic equipped with high-fidelity manikins, VR technology, and medical resources where students can practice clinical skills in a safe, structured space.
Self-Directed Learning & Choice	A student chooses which letter they want to practice writing and determines the duration of their work cycle without being dictated to by a rigid schedule.	A medical student identifies a personal knowledge gap in cardiology and utilizes asynchronous digital resources or selects specific electives to master that competency determining their duration of practice and work cycle.
Role of the Teacher as a Guide	The teacher observes the child’s progress with the stencils and provides subtle redirection or a new challenge only when the child is ready for the next step.	A resident acts as a mentor, facilitating clinical reasoning and providing formative feedback during patient rounds rather than delivering a standard lecture.
Mixed-Age/Level Collaboration	An older child in the classroom who has mastered writing assists a younger student in holding a stencil correctly.	Senior residents or fourth-year medical students coaching first-year students in a Team-Based Learning (TBL) environment or during interprofessional education (IPE) exercises.
Control of Error (Self-Correction)	A wooden puzzle is designed so a piece only fits in one specific spot; if it doesn’t fit, the child knows they need to try again without the teacher pointing it out.	High-fidelity simulation manikins that “react” to a student’s intervention (e.g., heart rate drops if the wrong drug is given), allowing the student to recognize and correct their own clinical reasoning in real-time.

## Montessori and Adult Learning Theories

To situate Montessori principles within the context of adult learners, we briefly examine their alignment with established theories of adult education. While Montessori’s educational philosophy was originally designed for children, its principles of autonomy, individualized pacing, and reflective engagement have strong parallels to Mezirow’s transformative learning and Knowles’ andragogy [4, 65]. While the educational contexts differ, this connection demonstrates how Montessori principles can be applied to adult learning contexts. Mezirow [65] emphasized that transformative learning occurs when adults critically reflect on prior assumptions and take ownership of their learning—a process Montessori initiates early through self-directed, choice-driven activities within a carefully prepared environment. Although Montessori did not use the language of critical reflection, her focus on observation, repetition, and internal motivation cultivates the metacognitive skills foundational to later adult reflection.

Similarly, Knowles’ theory of andragogy asserts that adult learners are most motivated when learning is self-directed, problem-centered, and immediately relevant to their personal and professional development [4, 65]. Montessori’s structured yet flexible approach anticipates these needs by encouraging learners to pursue knowledge aligned with their interests and developmental stage. In medical education, when learners must manage complex clinical information, reflect on ethical dilemmas, and respond to patients’ diverse needs, combining Montessori’s child-centered foundations with adult learning principles offers a powerful framework. Together, these theories underscore the importance of designing curricula and environments that support autonomy, relevance, reflection, and developmental readiness, which are core elements in preparing adaptive, self-aware healthcare professionals.

## Results

Applying principles from our MIME Framework and adult learning theories to medical education can significantly enrich physician training by fostering a learner-centered, reflective, and ethically grounded approach. In Montessori philosophy, the teacher guides inquiry and practice while the student is an active, self-motivated learner, and the environment is intentionally prepared to promote exploration and mastery [2]. The Montessori principle of “respect for the learner” promotes a more humanistic and inclusive learning environment, one that prioritizes student agency and well-being. This same respect naturally extends to patient care, reinforcing the patient-centered ethos that underpins modern healthcare. These elements offer a cohesive and innovative approach to cultivating competent, compassionate, and reflective physicians and provide meaningful insights for designing medical curricula that foster independence and clinical competence [18, 53].

A study of self-directed learning (SDL) in medical education by Hill et al. [53] suggested how how Montessori-inspired strategies may support the development of clinical knowledge and lifelong learning skills in first-year medical students. Students identified their knowledge gaps, researched credible sources, and collaborated on team-based reports. Students reported enhanced lifelong learning skills and greater confidence in clinical reasoning.

This example suggested how several foundational Montessori principles could be applied in a medical education setting. The activity supports learner autonomy, a cornerstone of Montessori philosophy, by encouraging students to identify and address their own knowledge gaps [66, 67]. Such activities exemplify how learner autonomy operates within defined boundaries—a concept central to both self-directed medical training and the Montessori approach. Meanwhile, the Montessori principle of “freedom within limits” finds a strong parallel in the ethical and professional frameworks of clinical training, which encompass patient safety, informed consent, professionalism, and interprofessional collaboration. This approach, which grants learners structured independence, is crucial for developing future healthcare professionals. For instance, in a clinical setting, a medical student might be given the freedom to propose a diagnostic workup for a patient, but within the limits of established protocols and under the supervision of an attending physician. This allows them to navigate complex decisions, balance their burgeoning clinical judgment with professional responsibility, and develop internal accountability—all while ensuring patient safety.

Beyond structured independence, the cultivation of an internal motivation is central to both Montessori principles and medical education. The Montessori learning environment emphasizes that for learning to be truly effective and sustained, it must be rooted in intrinsic motivation. Similarly, medical education prioritizes authentic curiosity. When students are genuinely invested in understanding complex medical concepts and honing their skills to become competent healers, they engage more deeply with the material. This intrinsic drive fosters the development of essential qualities for effective medical practice, such as compassion, empathy, and a sustained desire for continuous learning and growth, rather than simply performing tasks for grades or recognition. Integral to this engagement is the principle of individualized progression, another cornerstone of Montessori education. Loeffler [2] suggests that Montessori’s emphasis on adapting didactic materials based on observational feedback promotes a flexible and responsive learning approach. Medical education is shifting away from “one-size-fits-all” models toward competency-based medical education (CBME), a similarly flexible curricula that respond to individual learning needs and allow students to progress based on demonstrated competency rather than fixed timelines [64, 68]. This competency-based structure aligns with Montessori’s emphasis on repetition and self-directed mastery, encouraging learners to engage in iterative skills practice and self-assessment until achieving proficiency [18]. The emphasis on observational assessment and formative feedback supports coaching-based models and encourages reflective practice, which are increasingly recognized as essential to professional development in healthcare [69]. Assessment practices grounded in continuous observation and self-reflection may provide more nuanced feedback and support personalized learning pathways.

Lastly, the concept of a prepared environment, which is an intentionally organized space that supports learner autonomy and exploration translates well into medical education contexts. In Montessori classrooms, materials are carefully selected and arranged to allow students to choose meaningful tasks independently [2]. In a medical education context, this concept could translate into a psychologically safe and supportive setting such as simulation labs, clinical learning environments, and resources that promote self-directed, experiential learning. This environment enables students to actively engage with learning and develop the habits of self-directed inquiry essential for lifelong learning in medicine. Loeffler [2] emphasizes the role of educators in cultivating environments that invite curiosity and provide the structure necessary for learners to thrive. In other words, by preparing environments that foster reflection, clinical skills, and patient empathy, medical education can create learning experiences that align with the cognitive and emotional development required in clinical practice.

## Discussion

### Curricular Implications: Alignments and Challenges in Medical Education

While contemporary medical education has increasingly embraced elements of learner-centered and competency-based approaches, several core aspects of Montessori education remain underutilized. Integrating these elements could significantly enhance the development of ethical, empathetic, and lifelong learners in medicine.

The Montessori idea of “freedom within limits” aligns well with the ethical frameworks in clinical training (e.g., beneficence, autonomy, justice). By allowing learners structured independence, students learn to navigate complex decisions, balance clinical judgment with professional responsibility, and develop internal accountability. Central to this independence is intrinsic motivation, which fosters deeper engagement with content and supports the development of compassion, empathy, and a sustained desire for growth, qualities vital for effective medical practice. However, medical training often fragments this process with rigid schedules, frequent evaluations, and performance-driven assessments. This structure may inhibit the development of internal motivation and reflection [70]. Montessori’s emphasis on uninterrupted work cycles, where learners engage deeply in self-chosen tasks without external time constraints, offers protected time for exploration and self-guided study. Allowing learners this time could cultivate deeper cognitive engagement, intrinsic motivation, and metacognitive awareness, skills critical for continuous professional development [71, 72].

Another Montessori principle found in limited aspects of medical education is multi-age collaboration, where students of different levels work together. This collaboration allows novices to learn from more experienced peers while advanced learners reinforce their knowledge through teaching. While peer learning exists in some clinical settings, formal structures that promote longitudinal mentoring and reciprocal peer education remain limited. Integrating more peer-to-peer models could build connectedness, another key element for motivation [70], as well as foster humility, collegiality, and empathy, traits essential to ethical and team-based healthcare.

Whereas multi-age collaboration values peer interdependence, Montessori’s profound respect for the learner’s development trajectory runs counter to the standardized pathways of medical education. Unlike Montessori’s individualized progression, most medical education curricula adhere to rigid time-based milestones. By shifting to truly personalized competency-based models, institutions could support learners in developing not only competence but also self-regulation, resilience, and ethical clarity [73].

Finally, Montessori’s emphasis on grace and courtesy, deliberate instruction in respectful, mindful interaction, are seldom explicitly taught in medical programs, even though these skills speak directly to the relational and ethical competencies required of medical professionals. Incorporating structured reflection on professional behavior, empathy, and relational ethics could better prepare students to become compassionate healers attuned to the human dimensions of care [74, 75].

Despite the alignment between several Montessori principles and medical education, implementing the Montessori Method into medical school curricula that are highly regulated, time-restricted, and exam-oriented presents a significant challenge.

The current generation of medical students is a heterogeneous group, including not only recent college graduates but also individuals who have taken gap years, worked in clinical or research roles, or earned other advanced degrees. This demographic is also notably familiar with virtual and asynchronous learning, largely due to the widespread adoption of digital learning technologies and the educational shifts brought on by the COVID-19 pandemic. The familiarity of these learners with new content delivery mechanisms offers an opportunity to explore self-paced curriculum models in the pre-clerkship years. This approach could shift the role of faculty from traditional lecturers to facilitators, guiding students through their own learning journeys. However, a self-paced model faces several obstacles. Logistical challenges abound, particularly in managing a large cohort of students (often over 100) who are progressing at different rates. In addition, students who fall behind may face academic setbacks, potentially needing to repeat a year to catch up. Perhaps the most significant challenge lies in the fundamental conflict between Montessori principles and the assessment-driven nature of medical education. While the Montessori Method emphasizes self-directed, lifelong learning [76], medical curricula require students to pass high-stakes assessments at specific points to advance. This outcome-driven focus creates a barrier to a truly self-paced model, as it necessitates a uniform pace for progression. Therefore, adopting a curriculum that incorporates many Montessori principles would require overcoming substantial institutional and logistical hurdles.

While the direct application of the Montessori Method to medical education remains largely unexplored in academic literature, our study identifies significant conceptual overlaps between its core principles and certain aspects of medical school curricula. The fundamental emphasis of the Montessori approach on self-directed, hands-on learning and the role of the educator as a facilitator aligns well with the evolving landscape of medical education. The increasing use of asynchronous, virtual content delivery and a diverse student body that is comfortable with distributed learning technologies presents a natural opportunity to integrate more self-paced learning modules. This evolution could shift faculty from a front-facing lecturing role to a more guiding, mentorship-based position, a model consistent with Montessori philosophy.

### Future Pathways

Daisy Han’s “Montessori for Adults: How Do We Raise Innovators?” [77] offers medical educators a valuable framework for addressing common curriculum challenges by emphasizing adaptable principles like cultivating curiosity, fostering innovation, and promoting collaborative learning. While initially aimed at adult learners within a broader educational context, these insights provide a clear path to enriching medical education beyond traditional, rigid structures.

The principle “cultivating curiosity” highlights the importance of fostering a culture of inquiry [77]. In the medical education context, learning experiences could encourage medical students to question established practices and seek to understand the evolving dynamics of patient care. Fostering curiosity could help students become more adaptable and open-minded clinicians, prepared to engage with new challenges as they arise. Whereas the idea of trusting yourself to innovate can inspire the medical curriculum to encourage students to explore various approaches to clinical problem-solving and patient interaction.

Another key principle, “collaborative learning,” emphasizes the importance of structuring learning environments that promote teamwork and shared knowledge [77], which can prepare medical students for the interprofessional nature of healthcare. Consequently, cross-disciplinary learning can further support this collaborative spirit and prepare students better to work interprofessionally. Together, these Montessori-inspired principles bolster core competencies like critical thinking and adaptability, enhancing the development of future healthcare professionals.

Cross-disciplinary engagement also allows medical educators to build curricula that are not only scientifically grounded but also responsive to learners’ social and emotional needs, essential for fostering a holistic approach to patient care. Loeffler [2] argues that Montessori educators are encouraged to engage with broader educational communities to adapt and refine Montessori’s ideas for contemporary challenges. This open, interdisciplinary approach can benefit medical educators, who increasingly recognize the value of integrating insights from fields such as psychology, education, and technology to enrich medical curricula.

However, the shift from theoretical benefits to evidence-based implementation is hindered by a significant gap in the literature. Little research exists on the effect of the Montessori Method on other models in K-12 education, nor is there a great deal of longitudinal research on the effects of a Montessori education [9, 78]. Because elements of the Montessori model have not been explicitly identified as such in higher education, especially in medical education, it may be difficult to measure the effect of any one principle trait on students’ training. Future research may want to explore how Montessori principles explicitly improve outcomes in medical education. Researchers struggle with measuring the success of the Montessori Method in primary and secondary education since it can be difficult to determine how faithful they are to Montessori principles [9, 76]; this struggle will likely be perpetuated in higher education programs that do not fit as part of the traditional Montessori curriculum. Another challenge in measuring the effectiveness of Montessori training lies in the tension between the goal of Montessori education - development of life-long, self-directed learning - and the outcome-based focus of medical education [9, 76].

While this paper distinguishes Montessori principles from Montessori education, critics may argue that applying these elements in alternative settings dilutes the purity of the Montessori tradition.

Finally, the MIME Framework can provide a foundation for empirical investigation across multiple study designs as follow:

- *Design-based implementation research (DBIR) approach* could be used to redesign a pre-clerkship block as a foundational biomedical sciences using MIME as the guiding heuristic. In this model, successive cycles of curricular refinement would align structural elements such as time allocation and coaching systems with instructional processes, and learner autonomy to support targeted outcomes. while collecting and implementing performance data to monitor necessary adjustment.
- *Mixed-methods evaluation could examine the relationship between MIME-aligned redesign and learner outcomes* by integrating quantitative measures such as autonomy-supported scales, self-directed learning readiness, reflective capacity instruments, workplace-based assessments with qualitative data such as focus groups exploring belonging, agency, and identity formation.
- *Comparative mapping of curricula across institutions* could benefit by a standardized MIME approach to analyze curricular design patterns, assess inter-rater reliability, and explore associations between Structure–Process–Freedom configurations and educational outcomes.

Together, these approaches position MIME not only as a conceptual model but as a testable framework for educational scholarship and cross-institutional inquiry.

## Data Availability

Data sharing not applicable to this article as no datasets were generated or analysed during the current study.

## Declarations

### Disclaimer

The opinions or assertions contained herein are the private ones of the authors/speakers and are not to be construed as official or reflecting the views of the Department of Defense, the Uniformed Services University of the Health Sciences (USU), or any other agency of the U.S. government.
